# Malignant Pancreatic Polypeptide Secreting Tumour of Islet Cells: A Case for Aggressive Surgical Palliation

**DOI:** 10.1155/1993/20740

**Published:** 1993

**Authors:** R. D. Pullan, M. W. Scriven, J. O'dowd, A. T. Edwards, M. H. Lewis

**Affiliations:** Departments of Surgery and Pathology East Glamorgan General Hospital Church Village Mid Glamorgan Wales CF38 lAB USA

## Abstract

A case of a malignant pancreatic polypeptide secreting tumour is reported. The tumour was metastatic at presentation at which time it was excised. Pancreatic duct obstruction occurred 3 years after excision causing severe pain on eating. Major palliative surgery, in the form of a pancreatico-jejunostomy, cured the severe symptoms. The patient survives, largely symptom free, over six years after original excision. This case illustrates the need for aggressive management of symptoms in tumours in which long term survival is possible despite locally advanced or metastatic disease.

ABBREVIATIONS: VIP — vasoactive intestinal peptide. CT — computed tomography. GI — gastrointestinal. HPP — human pancreatic polypeptide. APUD — amine precursor uptake and decarboxylation.


*HPB Surgery*, 1993, Vol. 6, pp. 301–309
Reprints available directly from the publisher
Photocopying permitted by license only

© 1993 Harwood Academic Publishers GmbH
Printed in the United States of America

### MALIGNANT PANCREATIC POLYPEPTIDE SECRETING TUMOUR OF ISLET CELLS: A CASE FOR AGGRESSIVE SURGICAL PALLIATION

R.D. PULLAN, M.W. SCRIVEN, J.O'DOWD,* A.T. EDWARDS and M.H. LEWIS

*Departments of Surgery and Pathology*, East Glamorgan General Hospital, Church Village, Mid Glamorgan CF38 1AB, Wales*

*(Received 29 January 1993)*

A case of a malignant pancreatic polypeptide secreting tumour is reported. The tumour was metastatic at presentation at which time it was excised. Pancreatic duct obstruction occurred 3 years after excision causing severe pain on eating. Major palliative surgery, in the form of a pancreatico-jejunostomy, cured the severe symptoms. The patient survives, largely symptom free, over six years after original excision. This case illustrates the need for aggressive management of symptoms in tumours in which long term survival is possible despite locally advanced or metastatic disease.

ABBREVIATIONS: VIP — vasoactive intestinal peptide. CT — computed tomography. GI — gastro-intestinal. HPP — human pancreatic polypeptide. APUD — amine precursor uptake and decarboxylation.

KEY WORDS: Pancreatic apudoma, pancreatico-jujunostomy

### INTRODUCTION

Pancreatic islet cell tumours are rare. They are often associated with syndromes characteristic of excess of individual peptide hormones, such as gastrin, insulin, glucagon and vasoactive intestinal peptide (VIP)[1-3].

Amongst these tumours malignant pancreatic polypeptide (HPP) producing tumours are uncommon, and despite markedly elevated hormone levels may be asymptomatic[4,5]. They may thus present with advanced or metastatic disease.

We report a case of such a tumour, and review the management problems.

### CASE HISTORY

The patient, a 32 year old male, presented with a three week history of right upper quadrant pain, diarrhoea, abdominal distension and flatulence. His bowel motions were offensive smelling and of a slightly loose but otherwise normal consistency. There was a history of heavy smoking and alcohol abuse. The history was otherwise normal.

Address correspondence to: Mr Lewis at the above address.

General examination revealed a normal looking man with no lymphadenopathy nor jaundice and normal vital signs. A 7 by 5 cm mass was palpable in the right upper quadrant. This was tender and distinct from the liver. Rectal examination was normal although the stool was pale.

Routine haematological and biochemical tests, including liver function tests, were normal, as was urinalysis. A GI (gastro-intestinal) hormone profile was not performed at this time.

Plain radiographs of the abdomen showed a soft tissue shadow consistent with the palpable mass. Ultrasonography of the mass showed it to be solid (8 x> 10 cm) with a central cystic area. It was separate from the right kidney and the head of the pancreas. The scan was otherwise normal. Barium enema showed a normal colonic mucosa with extraluminal compression of the ascending and transverse colon. There was no obstruction. These appearances were felt to be consistent with a mesenteric neoplasm. Intravenous urography was normal.

The patient underwent exploratory laparotomy at which a 12 by 10 cm oval mass was found to be occupying the greater part of the transverse mesocolon. It appeared to arise from the head of the pancreas to which it was adherent. Laparotomy, in particular inspection of the liver and the rest of the pancreas, was otherwise normal. The mass was resected with a margin of normal pancreas.

### HISTOLOGY

Macroscopically the tumour was composed of firm, grey tissue showing focal haemorrhage and weighed 400 g.

Microscopically the tumour cells were arranged in nests and ribbons with an intervening vascular supporting stroma (Figure 1). The nuclei were fairly uniform being vesicular with small nucleoli. Intermingling with the tumour, at its edge, were non-neoplastic pancreatic ducts and acini, supporting an origin from the pancreas. A Grimelius stain was positive for agyrophil granules. Whilst the main lesion had clear resection margins metastatic tumour was seen in one adjacent lymph node.

Immunohistochemical stains showed areas within the tumour to be positive for gastrin, vasoactive intestinal peptide, pancreatic polypeptide, somatostatin and glucagon.

The histological appearances were of a malignant islet cell tumour of the pancreas.

### POST OPERATIVE COURSE

The post-operative recovery was rapid and uneventful. The only residual symptom was diarrhoea up to six times a day.

A number of investigations were undertaken post operatively. Blood gastro-intestinal (CI) hormone levels were measured and were all normal except for HPP which was over 1000 pmol/l (normal < 300). CT scanning showed a normal pancreas with a poorly enhancing area in each side of the liver. These were felt to be metastases, although they had not been palpable nor visible at operation. As the patient was asymptomatic no further treatment was undertaken and the patient was

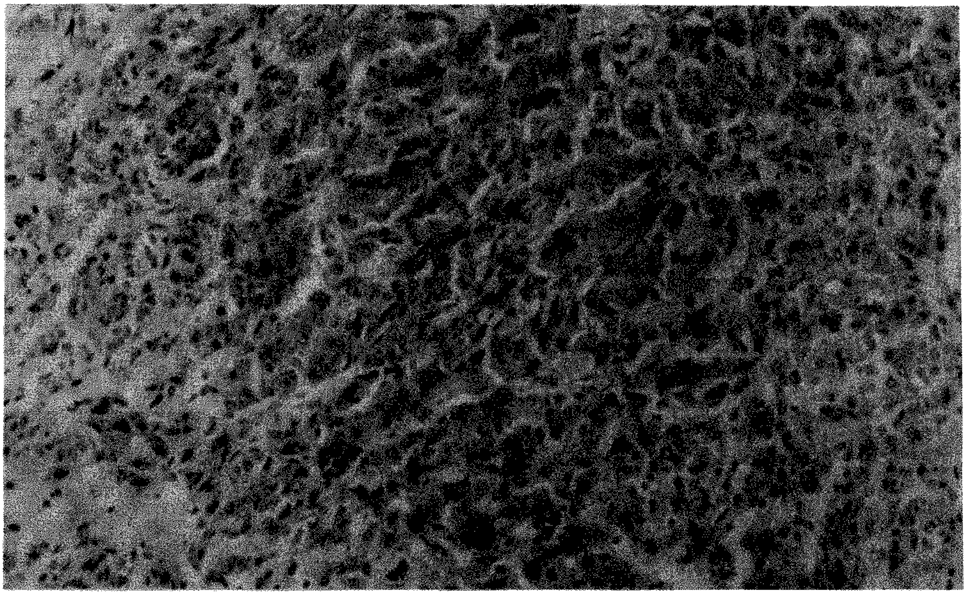

**Figure 1** The original tumour, composed of clusters of cells with vesicular nuclei and prominent nucleoli (H & E × 100).

followed up in the outpatient clinic. This follow up included serial GI hormone levels and liver function tests. The patient remained well until 34 months post operatively.

### RE-PRESENTATION

At 34 months post operatively the patient reported severe epigastric and back pain, which came on about 15–30 minutes after eating. Examination at this time was normal.

Blood HPP was 17035 pmol/l (normal < 300) and neurotensin 183 pmol/l (normal < 100). The other GI hormones were within normal limits. Upper GI endoscopy and barium meal were normal.

Ultrasound and CT scanning showed a dilated pancreatic duct (2 cm in diameter) with a normally enhancing rim of pancreatic tissue. The ductal dilatation was maximal in the head of the pancreas. There was only slight if any dilatation of the extrahepatic biliary tree. A 4 cm diameter mass lay between the aorta, superior mesenteric vessels and the third part of the duodenum. Two small hypodense lesions were seen in the liver on CT but not on ultrasonography. Endoscopic retrograde cholangio-pancreatography failed to visualise the pancreatic duct but there was no abnormality of duodenum, papilla nor biliary tree.

The patient thus underwent a second laparotomy. Multiple nodules were found in each side of the liver, these were biopsied. No discrete mass was found in the area of the pancreas but there were dense adhesions from the previous surgery surrounding the head of pancreas. No other intraperitoneal tumour was identified. The pancreatic duct was needled and an on table pancreatogram performed (Figure 2). This showed the duct to be dilated to approximately 2 cm, down to the ampulla of Vater. A side-to-side pancreatico-jejunostomy was thus formed. The patient recovered quickly from this operation with no residual pain. Histological examination of the liver biopsies confirmed the clinical impression of metastatic tumour, of similar appearance to the original endocrine primary (Figure 3). Fine needle aspiration cytology of the pancreas showed no evidence of recurrent carcinoma.

When last seen in the outpatient clinic, 46 months after the second operation (80 months after the first) the patient was well and pain free. Loose bowel actions occur about six times daily but there are no other residual symptoms and his weight is steady. The serum HPP was over 10000 pmol/l and neurotensin over 1000 pmol/l.

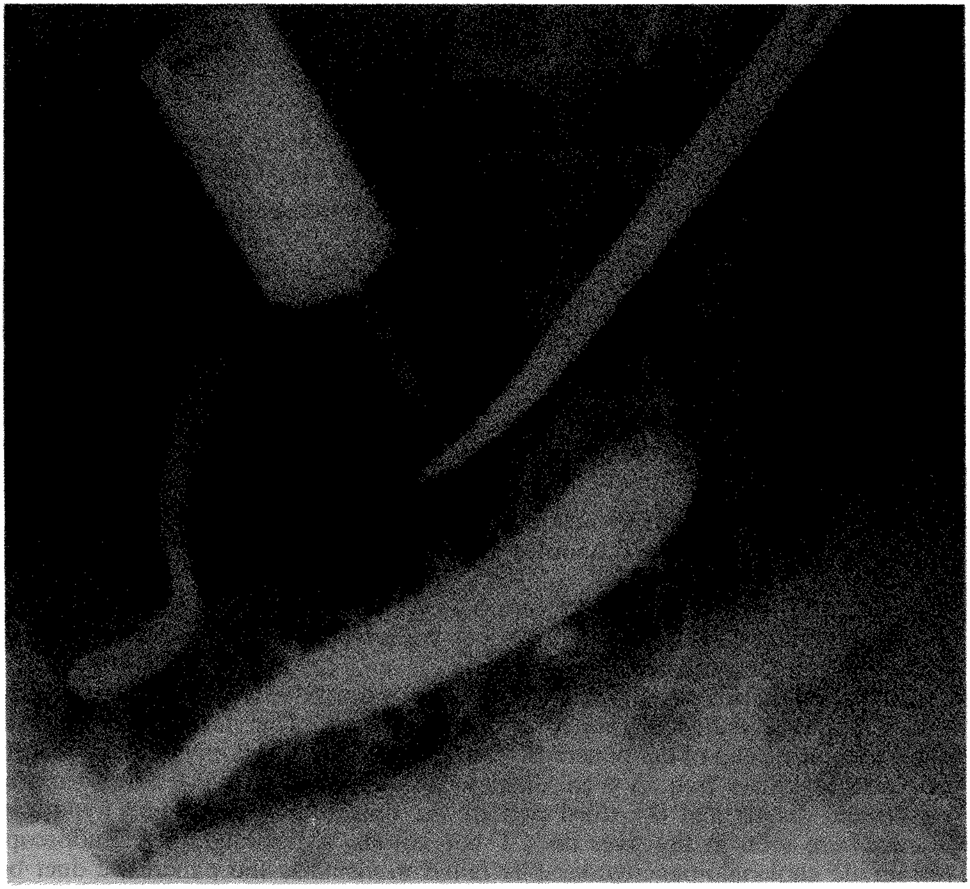

**Figure 2** Per-operative pancreatogram showing dilated pancreatic duct with a normal calibre distal common bile duct.

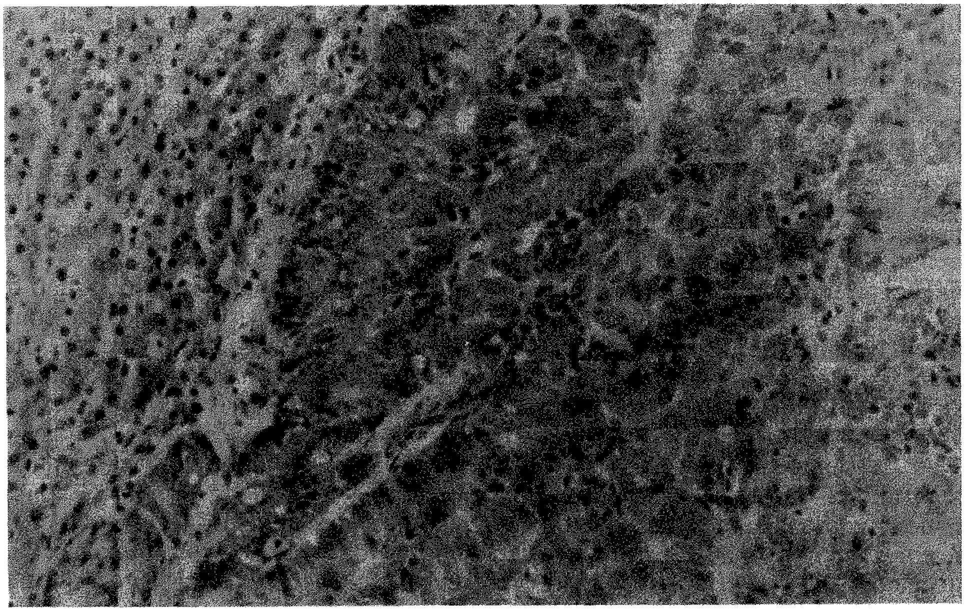

**Figure 3** Liver metastasis biopsied at the second laparotomy, showing ribbons of endocrine cells similar to the original tumour.

### DISCUSSION

Malignant pancreatic HPP secreting tumours are rare. The case reported illustrates many features that have been previously described and some that have not.

The pancreatic islets are found from cells of the APUD system. Tumours of these cells may present in a similar way to other pancreatic tumours, in particular with extrahepatic biliary obstruction or metastatic spread. However, they typically secrete peptide hormones in excess, often of more than one type[7]. The clinical manifestation of this excess depends on the predominant hormones.

HPP at present seems to have a relatively minor role in normal physiology and excess HPP may occur without clinical manifestation. Patients may thus present with locally advanced or metastatic disease[8]. This perhaps reflects the minor physiological role of HPP. HPP secreting tumours are associated with type 1 multiple endocrine neoplasia (MEN-1)[4], which is not so with other islet cell tumours[2]. Measurement of plasma HPP has been suggested as a marker for MEN-1[9] but the specificity is low[10] and can be affected by many other factors. Inspite of this it may well have a role as an adjunctive marker[11].

Excess HPP may, however, produce a syndrome similar to that of VIP excess — watery diarrhoea syndrome[12]. The diarrhoea, as our case illustrates, is not usually profuse or watery. This probably reflects a different mode of action from VIP. Unfortunately in our case pre-excision GI hormone levels are not available to show whether VIP was raised pre-operatively. The persistence of diarrhoea in the absence of raised VIP seems to refute this possibility.

Histology of the tumours shows the features typical of endocrine tumours. Immunohistochemical techniques allow the identification of hormones in the tumour. Typically a wide range of peptides are identified even though only one or two are secreted in excess[13]. This was clearly seen in our case.

Serial measurement of hormone profiles is often undertaken as part of follow up. By doing so other authors have reported co-secretion of neurotensin later in the progress of the disease. There is however only one reported case of this occurring with an HPP secreting tumour[14]. As with other reported cases[15–17] VIP was also secreted in this patient. This phenomenon occurred in our patient in the absence of excess VIP secretion.

The natural history of HPP secreting tumours is largely unknown. About half of reported cases are malignant[4, 18–21]. Metastases are usually hepatic, and less commonly lymph node. Long term survival is reported despite metastatic disease. Our case clearly demonstrates that despite proven lymph node metastasis (not direct spread into the lymph node) and probable hepatic metastases survival over six years is possible. This survival was with a very good quality of life.

As the tumour is so rare very little is known about the ideal form of treatment. In reported cases treatment of the primary tumour is usually by surgery although tumour embolisation or streptozotocin are other possibilities[20]. Treatment with streptozotocin can, however be associated with severe side effects and may not be justified. Long term survival in the presence of advanced disease means that treatment of local effects of the primary must be aggressive. The case reported herein shows that pancreatic decompression surgery is beneficial when pancreatic duct obstruction occurs. Our patient is perfectly well and pain free nearly four years after such surgery. Prior to this operation his pain, following eating, was so severe that he lost weight and the pain prevented him sleeping normally.

Treatment of metastatic or recurrent tumours must also take account of the possibility of long term survival. Certainly our case illustrates that symptom free liver metastasis can be associated with survival in excess of six years.It seems inappropriate to recommend resection of such metastatic hepatic tumours, especially when present in both lobes of the liver.

In summary we report a case of an HPP secreting pancreatic islet cell tumour. This tumour can be associated with long term survival despite localy advanced and metastatic disease. Treatment should therefore be aggressive in the case of symptomatic disease but expectant otherwise. The aggressive surgical management of painful pancreatic duct obstruction in our case has resulted in prolonged survival with excellent quality of life. Such treatment must thus be recommended.

### *References*

1. Friesen, S.R. (1982) Tumours of the endocrine pancreas. *N. Engl J. Med.*, **306**, 580–590
2. Wood, S.M., Polak, J.M. and Bloom, S.R. (1983) Gut hormone secreting tumours. *Scan. J. Gastroenterol*, Suppl **82**, 165–179
3. Gower, W.R. and Fabri, P.J. (1990) Endocrine neoplasms (non-gastrin) of the pancreas. *Semin. Surg. Oncol.*, **6**, 98–109
4. Vinik, A.I., Strodel, W.E., Eckhauser, F.E., Moattari, A.R., Lloyd, R. *et al.* (1987) Somatostatinomas PPomas neurotensinomas. *Semin. Oncol.*, **14**, 263–281
5. Strodel, W.E., Vinik, A.I., Lloyd, R.V. *et al.* (1984) Pancreatic polypeptide producing tumours: Silent lesions of the pancreas? *Arch. Surg.*, **119**, 508–514
6. Greenberg, G.R., McCloy, R.R. and Adrian, T.E. (1978) Inhibition of pancreas and galbladder by pancreatic polypeptide. *Lancet*, **2**, 1280–1282

7. Pearse, A.G.E. (1980) APUD concept and hormone production. *Clin. Endocrinol. Metabol.*, **9**, 211–222
8. Nobin, A., Berg, M., Ericsson, M., Ingemansson, S., Olsson, E. and Sundler, F. (1984) Pancreatic polypeptide producing tumours. A report of two cases. *Cancer*, **53**, 2688–2691
9. Friesen, S.R., Tomita,T., Kimmel, J.R, Doull, V. and Pollock, H.G. (1983) Pancreatic polypeptide update: its role in detection of the trait for multiple endocrine adenopathy syndrome type 1 and pancreatic polypeptide secreting tumours. *Surgery*, **94**, 1028–1037
10. Langstein, H.N., Norton, J.A., Chiang, H.C.V., O'Dorisio, Maton, P.N., Marx, S.J. and Jensen, R.T. (1990) The utility of circulating levels of human pancreatic polypeptide as a marker for islet cell tumours. *Surgery*, **108**, 1109–1116
11. Adrian, T.E., Uttenthal, L.O., Williams, S.J. and Bloom, S.R. (1986) Secretion of pancreatic polypeptide in patients with ancreatic endocrine tumours. *New Eng. J. Med.*, **315**, 287–291
12. Larsson, L.I., Schwartz, T. and Lundquist, G. (1976) Pancreatic polypeptide in pancreatic endocrine tumours. Possible implication in watery diarrhoea syndrome. *Am. J. Pathol.*, **85**, 675–684
13. Heitz, P.U., Kasper, M., Polak, J.M. and Kloppel, G. (1982) Pancreatic endocrine tumours. *Human Pathol.*, **13**, 263–271
14. Shulkes, A., Boden, R., Cook, J., Gallagher, N. and Furness, J.B. (1984) Characterization of a pancreatic tumour containing VIP, neurotensin and pancreatic polypeptide. *J. Clin. Endocrinol. Metabol.*, **58**, 41–48
15. Blackburn, A.M., Bryant, M.G., Adrian, T.E. *et al.*, (1981) Pancreatic tumours produce neurotensin. *J. Clin. Endocrinol. Metabol.*, **52**, 820–822
16. Bloom, S.R., Lee, Y.C., Lacroute, J.M. *et al.* (1983) Two patients with pancreatic apudomas secreting neurotensin and VIP. *Gut*, **24**, 448–452
17. Feurle, G.E., Helmstaedter, V., Tischbirek, K. *et al.* (1981) A multi-hormonal tumor of the pancreas producing neurotensin. *Digest Dis. Sci.*, **26**, 1125–1133
18. Martensson, H., Bottcher, G., Sundler, F. and Nobin, A. (1990) Localization and peptide content of endocrine pancreatic tumours. *Ann.Surg.*, **212**, 607–614
19. Choksi, U.A., Sellin, R.V., Hickey, R.C. and Samaan, N.A. (1988) An unusual skin rash associated with pancreatic polypeptide tumor of the pancreas. *Ann. Int. Med.*, **108**, 64–65
20. Friesen, S.R., Stephens, R.I. and Huard, G.S. (1981) Effective streptozocin therapy for metastatic pancreatic polypeptide apudoma. *Arch. Surg.*, **116**, 1090–1092
21. Tomita, T., Friesen, S.R., Kimmel, J.R., Doull,V. and Pollock, H.G. (1983) Pancreatic polypeptide secreting islet cell tumours: A study of three cases. *Am. J. Pathol.*, **113**, 134–142

*(Accepted by M. Puntis 24 January 1993)*

### INVITED COMMENTARY

Although islet cell tumours of the pancreas are rare, they may produce dramatic symptoms and signs as a result of their ability to secrete a variety of hormones and peptides. Approximately half of all islet cell carcinomas are nonfunctioning[1] and are often diagnosed serendipidously by CT scanning. Some tumours may be functioning but silent in that the excess secretion of hormone does not give rise to a recognisable clinical syndrome. Pancreatic polypeptide (PP) secreting tumours may fall into this category.

This polypeptide containing 36 aminoacids (molecular weight 4240 daltons) was discovered by Dr Kimmel in Kansas almost by chance whilst preparing insulin extracts from chicken pancreas[2,3]. The peptide was subsequently isolated from human pancreas[4]. The precise physiological role of PP is not certain, although its release is stimulated by a meal and inhibited by muscarinic cholinergic blockade[5].

This latter observation has been used as the basis of a test to distinguish between autonomous PP secretion by tumour and PP release by normal cells which can be blocked by atropine[6]. Co-secretion of PP by other tumours is well recognised and indeed there has been considerable support for the concept of using PP as a tumour marker, particularly in the context of the MENI syndrome[7]. Although the pancreatic apudomas in MENI are invariably multiple, it is of interest that the PP audomas can occasionally be single[8], an observation which has important therapeutic implications. Co-secretin of PP is seen in 50% of pancreatic endocrine tumours and is most common in association with vipomas and glucagonomas[9]. Raised pancreatic polypeptide levels have also been noted in the carcinoid syndrome[5] but do not occur in adenocarcinoma of the pancreas[10].

Pure PP secreting tumours are rare and there are few reported in the literature[11]. Because of the preponderance of PP secreting F (D2, PP) cells in the pancreatic head, it is not surprising that most tumours are found in this region. Most PPomas are malignant and have metastasised to the liver by the time of diagnosis. The present case reported by Lewis and colleagues is a good example and illustrates many of these characteristics. Diagnosis may be difficult in this often silent tumour but abdominal pain, mass, obstructive jaundice, hepatomegaly, splenic vein thrombosis, gastrointestinal haemorrhage, diarrhoea and a scaling erythematous skin rash[12] have all been described. The cause of diarrhoea is controversial but the suggestion that PP is responsible has been largely discredited[13]. In tumours with a mixed secretory pattern VIP may be the dominant hormone although PP is co-secreted in approximately 75% of VIPomas[14]. Under these circumstances VIP is likely to play a crucial role in the genesis of diarrhoea. In the present case report however it seems unlikely that VIP had any significant part to play. Other possible explanations for the diarrhoea have included steatorrhoea and pancreatic duct blockage[13]. The patient described by Lewis did have pancreatic duct obstruction but the diarrhoea continued unabated after the second operation to relieve the obstruction and decompress the duct.

It is known that neurotensin, a 13 aminoacid polypeptide can be co-secreted with VIP and occasionally PP. It has been suggested that neurotensin might actually stimulate PP release[15]. The present case would appear to be unique in that the tumour secreted neurotensin and PP, probably without co-secretion of VIP.

Clearly the interrelationships and complexity of hormonal co-secretion, with more than one hormone often being produced by a single cell, contribute to the difficulties in diagnosis and understanding of pancreatic apudomas. Traditionally immunocytochemistry has been employed to demonstrate the hormones and peptides within the cell, but this technique provides information more closely related to hormone storage rather than production and secretion. It is hoped that the technique of *in situ* hybridisation, capable of demonstrating mesenger RNA specific for production of a particular peptide by a given cell, will provide additional valuable information and aid the elucidation of the disease mechanisms involved in these fascinating pancreatic tumours. Treatment of overt solitary PPomas is by surgical excision. This may be possible by local excision or enucleation as in the present case, but occasionally requires a more radical pancreatic resection[16]. The management of a patient with an elevated PP level without an immediately identifiable tumour is more difficult and some have advocated exploration in all cases[17], whilst others propose that localisation is paramount, using the techniques of venous sampling or arteriography before proceeding to surgery[5].

The present authors are to be congratulated on their excellent clinical management of this rare pancreatic tumour. I would totaly agree with their view that aggressive surgical management of the local disease,, perhaps even by multiple surgical procedures is worthwhile and may be associated with good survival even in the presence of hepatic metastases. Resection of liver metastases, chemotherapy with streptozotocin and the use of somatostatin analogue may all play a role in the management of disseminated disease but would not seem to be appropriate in this particular case.

*References*

1. Thompson, G.B., van Heerden, J.A. Grant, C.S. Carney, J.A. and Ilstrup, D.M. (1988) Islet cell carcinomas of the pancreas: A twenty year experience. *Surgery*, **104**, 1011–1017
2. Kimmel, J.R., Pollock, H.G. and Hazelwood, R.L. (1968) Isolation and characterization of chicken insulin. *Endocrinology*, **83**, 1323–1330
3. Langslow, D.R., Kimmel, J.R. and Pollock, H.G. (1973) Studies of the distribution of a new avian pancreatic polypeptide and insulin among birds, reptiles, amphibians and mammals. *Endocrinology*, **93**, 558–565
4. Chance, R.E. (1972) (Discussion) *Diabetes*, (Suppl 2) **21**, 536
5. Vinik, A.I., Strodel, W.E., Eckhauser, F.E., Moattari, A.R. and Lloyd, R. (1987) Somatostatinomas, PPomas, Neurotensinomas. *Seminars in Oncology*, **14**, 263–281
6. Adrian, T.E., Uttenthal, L.O., Williams, S.J. and Bloom, S.R. (1986) Secretion of pancreatic polypeptide in patients with pancreatic endocrine tumors. *New Engl. J. Med.*, **315**, 287–291
7. Friesen, S.R., Tomita, T. and Kimmel, J.R. (1983) Pancreatic polypeptide update: Its roles in the detection of the trait for multiple endocrine adenopathy syndrome, type I and pancreatic polypeptide-secreting tumors. *Surgery*, **94**, 1028–1037
8. Friesen, S.R. (1982) Tumors of the endocrine pancreas. *New Engl. J. Med.*, **306**, 580–590
9. Modlin, I.M. and Adrian, T.E. (1990) Hormones, neurotransmitters, and other humoral markers of endocrine tumors. In: *Surgical Endocrinology — Clinical Syndromes*, Ed. Friesen, S.R. and Thompson, N.W. Lippincott J.B. Comp., Philadelphia, pp. 25–33
10. Vinik, A.I., Strodel, W.E., Cho, K.J., *et al.* (1983) Localisation of hormonally active gastrointestinal tumors. In: Thompson N.W. Vinik A.I. (Eds) *Endocrine Surgery Update*. Orlando, F.L., Grune and Stratton, pp 195–218
11. Tomita, T., Friesen, S.R., Kimmel, J.R., Doull, V. and Pollock, H.G. (1983) Pancreatic polypeptide secreting islet cell tumors. A study of three cases. *Am. J. Pathol.*, **113**, 134–142
12. Choksi, U.A., Sellin, R.V., Hickey, R.C., Samaan, N.A. (1988) An unusual skin rash associated with a pancreatic polypeptide producing tumor. *Annals Internal Med.*, **108**, 64–65
13. Shulkes, A., Boden, R., Cook, I., Gallagher, N. and Furness, J.B. (1984) Characterization of a pancreatic tumor containing vasoactive intestinal peptide, neurotensin and pancreatic polypeptide. *J. Clin. Endocrinol. Metab.*, **58**, 41–48
14. Long, R.G., Bryant, M.G. Mitchell, S.J. *et al.* (1981) Clinicopathological study of pancreatic and ganglioneuroblastoma tumours secreting vasoactive intestinal polypeptide, (vipomas) *Br. Med. J.*, **282**, 1767
15. Blackburn, A.M., Fletcher, D.R., Adrian, T.E., *et al.* (1980) Neurotensin infusion in man — pharmacokinetics and effects on gastrointestinal and pituitary hormones. *J. Clin. Endocrinol. Metab.*, **51**, 1257
16. Strodel, W.E., Vinik, A.I., Lloyd, R.V., Glaser, B. *et al.* (1984) Pancreatic polypeptide producing tumors. Silent lesions of the pancreas? *Arch. Surg.*, **119**, 508–514
17. Friesen, S.R., Kimmel, J.R. and Tomita, T. (1980) Pancreatic polypeptide as screening for pancreatic polypeptide apudomas in multiple endocrinopathies. *Am. J. Surg.*, **139**, 61–72

